# Effects of Genetic Loci Associated with Central Obesity on Adipocyte Lipolysis

**DOI:** 10.1371/journal.pone.0153990

**Published:** 2016-04-22

**Authors:** Rona J. Strawbridge, Helmut Laumen, Anders Hamsten, Michaela Breier, Harald Grallert, Hans Hauner, Peter Arner, Ingrid Dahlman

**Affiliations:** 1 Atherosclerosis Research Unit, Department of Medicine Solna, Karolinska Institutet, Stockholm, Sweden; 2 Chair of Nutritional Medicine, Else Kröner-Fresenius-Center for Nutritional Medicine and ZIEL-Research Center for Nutrition and Food Sciences, Technische Universität München, Munich, Germany; 3 Clinical Cooperation Group Nutrigenomics and Type 2 Diabetes, Helmholtz Zentrum Muenchen and Technische Universität München, Munich, Germany; 4 German Center for Diabetes Research (DZD), Neuherberg, Germany; 5 Institute of Experimental Genetics, Helmholtz Zentrum Muenchen, German Research Center for Environmental Health, Neuherberg, Germany; 6 Research Unit of Molecular Epidemiology, Helmholtz Zentrum Muenchen, German Research Center for Environmental Health, Neuherberg, Germany; 7 Institute of Epidemiology II, Helmholtz Zentrum Muenchen, German Research Center for Environmental Health, Neuherberg, Germany; 8 Else Kröner-Fresenius-Center for Nutritional Medicine, Klinikum rechts der Isar, Technische Universität Muenchen, Munich, Germany; 9 Department of Medicine, Huddinge, Karolinska Institutet, Stockholm, Sweden; GDC, GERMANY

## Abstract

**Objectives:**

Numerous genetic loci have been associated with measures of central fat accumulation, such as waist-to-hip ratio adjusted for body mass index (WHRadjBMI). However the mechanisms by which genetic variations influence obesity remain largely elusive. Lipolysis is a key process for regulation of lipid storage in adipocytes, thus is implicated in obesity and its metabolic complications. Here, genetic variants at 36 WHRadjBMI-associated loci were examined for their influence on abdominal subcutaneous adipocyte lipolysis.

**Subjects and Methods:**

Fasting subcutaneous adipose tissue biopsies were collected from 789 volunteers (587 women and 202 men, body mass index (BMI) range 17.7–62.3 kg/m^2^). We quantified subcutaneous adipocyte lipolysis, both spontaneous and stimulated by the catecholamine isoprenaline or a cyclic AMP analogue. DNA was extracted from peripheral blood mononuclear cells and genotyping of SNPs associated with WHRadjBMI conducted. The effects on adipocyte lipolysis measures were assessed for SNPs individually and combined in a SNP score.

**Results:**

The WHRadjBMI-associated loci *CMIP*, *PLXND1*, *VEGFA* and *ZNRF3-KREMEN1* demonstrated nominal associations with spontaneous and/or stimulated lipolysis. Candidate genes in these loci have been reported to influence NFκB-signaling, fat cell size and Wnt signalling, all of which may influence lipolysis.

**Significance:**

This report provides evidence for specific WHRadjBMI-associated loci as candidates to modulate adipocyte lipolysis. Additionally, our data suggests that genetically increased central fat accumulation is unlikely to be a major cause of altered lipolysis in abdominal adipocytes.

## Introduction

With an almost epidemic global increase in obesity [1], related complications including type 2 diabetes and cardiovascular disease are a growing burden on healthcare systems. However, it has become clear that the distribution of fat tissue and the function of adipose tissue, rather than the amount of fat *per se*, exert an important impact [2, 3]. In particular, central fat accumulation, reflected by high waist-to-hip ratio (WHR), is strongly associated with risk for type 2 diabetes and other complications [3].

There is a strong genetic component influencing fat distribution. WHR adjusted for body mass index (WHRadjBMI) can be considered as a measure of fat distribution independent of overall obesity [4]. Recent genome wide association studies (GWAS) have identified several genetic loci associated with WHRadjBMI [4–6]. These findings notwithstanding, the identity of both the culprit genes in the loci and the mechanisms by which they influence adipose function or clinical phenotypes remain unclear. Importantly, whereas central regulation of food intake is believed to be a major determinant of overall obesity and BMI [7], it seems likely that fat distribution, as assessed by WHR, is primarily regulated locally in adipose tissue. In support of this, many genes at WHRadjBMI-associated loci are expressed in adipose tissue [8]. In addition, a few genes at these loci have recently been shown to be differentially expressed between subcutaneous and visceral adipose tissue compatible with a depot specific function [8].

A major function of adipose tissue is to store and release adipocyte fatty acids through esterification to triglycerides and hydrolysis of these triglycerides (lipolysis) according to the body’s energy demands. Disturbances in adipocyte lipolysis have been associated with abdominal obesity and WHR [9, 10]. Increased spontaneous lipolysis provides excess amounts of fatty acids which, in turn, induce insulin resistance [11, 12]. It is well established that the ability of catecholamines to stimulate lipolysis is blunted in obese individuals [13]. These subjects may retain lipids in adipocytes and thereby expand adipose tissue since the triglycerides within adipocytes constitute >90% of adipocyte volume and is the major volume component of the adipose mass.

Herein, we tested the hypothesis that genetic variation in WHRadjBMI-associated loci influence fat cell lipolysis. We utilized a unique large patient cohort in which both spontaneous and catecholamine-stimulated subcutaneous abdominal adipocyte lipolysis was measured.

## Subjects and Methods

### Cohort description

Characteristics of the study population are presented in Table 1. Adult subjects with a large inter-individual variation in body mass index (BMI, range 17.7–62.3 kg/m^2^, 587 women and 202 men) were recruited by local advertisement. All participants were examined at 8 a.m. after an overnight fast. A trained research nurse collected information about body weight, height, waist, and hip circumferences. A venous blood sample was obtained for routine laboratory measurements and extraction of DNA. Finally, a subcutaneous adipose tissue biopsy was obtained by needle aspiration under local anesthesia from the abdominal region. All subjects were of European origin, were healthy except for obesity, and were not taking any medication. For consistency with the previous reports [4–6], WHRadjBMI was calculated in a sex-specific manner by inverse-normal transformation of the residuals of the linear regression model: WHR adjusted for age, age^2^ and BMI [4]. The study was approved by the regional Ethics Committee in Stockholm and all subjects gave their written informed consent to participation.

**Table 1:** Cohort characteristics.

Sex	Men	Women
N	202	587
Age (years)	42 (14)	39 (10)
Height (m)	1.79 (0.07)	1.67 (0.07)
Waist (cm)	104 (20)	106 (21)
WHR	0.99 (0.07)	0.92 (0.09)
BMI (kg/m^2^)	29.6 (7.4)	33.8 (8.8)
Glucose (mmol/l)	5.38 (1.15)	5.26 (0.94)
Insulin (pmol/l)	12.7 (10.2)	11.6 (7.5)
Total cholesterol (mmol/l)	5.38 (1.34)	5.02 (1.05)
HDL cholesterol (mmol/l)	1.14 (0.33)	1.32 (0.36)

Where: WHR, waist:hip ratio; Values presented as mean (sd).

### Lipolysis experiments

The adipose tissue specimens were brought to the laboratory, rinsed repeatedly in saline and visual blood vessels and cell debris were removed. Spontaneous or unstimulated lipolytic activity was determined in explants of adipose tissue as described [12]. In brief, pieces of adipose tissue (200 or 300 mg) were incubated for 2 h as described below (100 mg/ml). Glycerol release into the medium was measured using a sensitive bioluminescence method and expressed as amount of glycerol release per 2 h and 10^7^ adipocytes [12]. Adipocytes are the only source of glycerol which is an end product of lipolysis.

The remaining tissue (about 1 gram) was subjected to collagenase treatment as described [14] to obtain isolated adipocytes. Measurement of stimulated lipolysis in isolated adipocytes was investigated as previously described [15]. Diluted cell suspensions (2% v/v) were incubated in duplicate for 2 h at 37°C with air as the gas phase in Krebs–Ringer phosphate buffer (pH 7.4) supplemented with glucose (8.6 mmol/l), ascorbic acid (0.1 mg/ml) and bovine serum albumin (20 mg/ml), without (basal) or with increasing concentrations (10^−9^–10^−5^ mol/l) of the synthetic non-selective β1, β2 and β3-adrenoreceptor agonist isoprenaline (Hässle, Mölndal, Sweden), or 10^−5^ to 10^−3^ mol/l of dibutyryl cyclic AMP (dcAMP, Sigma-Aldrich, Stockholm, Sweden), which is a phosphodiesterase-resistant cyclic AMP analogue that activates the cyclic AMP-dependent protein kinase A (S1 Fig). Variations in the lipolytic action of the non-metabolizing dcAMP can only be explained by variations in lipolysis activation at, or beyond, the level of cAMP-dependent protein kinase A, whereas lipolysis variation of isoprenaline can be explained at any step in catecholamine stimulated lipolysis, from beta-adrenoreceptors to hormone sensitive lipase [13]. Stimulated lipolysis was expressed as the ratio of isoprenaline-induced or dcAMP-induced amount of glycerol release per 2 h at maximum effective concentration divided by basal glycerol release. We recently demonstrated that this measurement correlates better with clinical phenotypes than expression of absolute rates of glycerol release per adipocyte [16, 17].

### Selection and genotyping of WHRadjBMI-associated single nucleotide polymorphisms

Single nucleotide polymorphisms (SNPs) tagging loci robustly associated with central fat accumulation as measured by WHRadjBMI were identified from literature [4–6]. Where the same locus is reported by multiple papers, the tag SNP was chosen from the first reported study. Due to the multiplex format used for genotyping, proxies were used to tag 3 SNPs: rs4531856 was used instead of rs12608504 (*JUND*) [6], rs1550280 instead of rs7830933 (*NKX2-6*) [6] and rs2165295 instead of rs1440372 (*SMAD6*) [6] (r^2^ = 1/0.96/1 respectively, 1000 Genomes pilot 1, CEU data [18]). Genotyping was conducted in two rounds: initially SNPs reported by Heid *et al* [4] and Randall *et al* [5], were analysed. Subsequently, SNPs reported by Shungin *et al* [6] were genotyped (with an additional 100 subjects being included). Genotyping of 48 WHRadjBMI-associated loci was conducted using the MassARRAY system with the iPLEX chemistry (Sequenom, San Diego, CA). Samples were analysed by matrix-assisted laser desorption-ionization time-of-flight (MALDI-TOF) mass spectrometry (Bruker Daltonik, Leipzig, Germany). MassARRAY TYPER 4.0 software (Sequenom) was used for peak identification within the mass spectra. One tagging SNP, rs9491696 (*RSPO3*, assay C___2789025_10), was genotyped using the Taqman system (Applied Biosystems, Stockholm, Sweden) according to the manufacturer’s instructions. Genotyping failed for tag SNPs in seven loci (*KCNJ2* rs8066985; *GORAB* rs10919388; *DCST2* rs905938; *KLF13* rs8042543; *HOXA11* rs7801581; *MACRD1-VEGFB* rs11231693 [6]; *CPEB4* rs6861681 [4]). The tag SNPs for 42 loci (Table 2) were successfully genotyped. For five loci, genotype frequencies deviated significantly from Hardy-Weinberg equilibrium (*SNX10* rs1534696; *NMU* rs3805389; *PEMT* rs4646404; *LY86* rs1294421; *ITPR2-SSPN* rs718314, (S1, Table)). Of these, two SNPs demonstrated very low heterozygous counts (*LY86* rs1294421 and *ITPR2-SSPN* rs718314), possibly indicative of technical issues with the assays, thus these SNPs were excluded from analysis. For three SNPs (*SNX10* rs1534696, *NMU* rs3805389 and *PEMT* rs4646404) the genotype frequencies were skewed but plausible, and possibly due to high rate of obesity in the studied cohort, thus these SNPs were included in analyses. Thus 40 tag SNPs were further analysed (Table 2). Where Shungin et al [6] report the same SNP as Heid et al [4], effect allele, effect allele frequency and effect sizes are given from Shungin et al due to the larger sample size. Of note, Randall *et al* [5] and Shungin *et al* [6] both report signals denoted as *MAP3K1*, however as the SNPs are >700Kb apart and in incomplete linkage disequilibrium (r^2^ = 0.88 between rs11743303 and rs9687846 [18]) they are both included in this report.

**Table 2:** Characteristics of genotyped SNPs tagging 40 of 49 known WHRadjBMI-associated loci.

CHR	SNP	Position	Locus^†^	First report	EA	Beta	EAF	Sex**
1	rs984222	119503843	*TBX15-WARS2*	Heid 2010	G	0.03	0.64	-
1	rs1011731	172346548	*DNM3-PIGC*	Heid 2010	G	0.03	0.43	-
1	rs4846567	219750717	*LYPLAL1*	Heid 2010	G	0.03	0.72	F
2	rs1385167	66200648	*MEIS1*	Shungin 2015	G	0.03	0.15	-
2	rs10195252	165513091	*GRB14-COBLL1*^‡^	Heid 2010	T	0.03	0.60	F
3	rs4684854	12488882	*PPARG*	Randall 2013	C	0.04	0.42	F
3	rs6784615	52506426	*PBRM1*	Heid 2010	T	0.04	0.94	-
3	rs6795735	64705365	*ADAMTS9*	Heid 2010	C	0.03	0.59	F
3	rs10804591	129334233	*PLXND1*	Shungin 2015	A	0.03	0.79	F
3	rs17451107	156797609	*LEKR1*	Shungin 2015	T	0.03	0.61	-
4	rs3805389	56482750	*NMU*	Shungin 2015	A	0.01	0.28	F
4	rs9991328	89713121	*FAM13A*	Shungin 2015	T	0.02	0.49	F
4	rs303084	124066948	*SPATA5-FGF2*	Shungin 2015	A	0.02	0.80	-
5	rs11743303	55859952	*MAP3K1*	Randall 2013	G	0.03	0.21	F
5	rs9687846	56566067	*MAP3K1*	Shungin 2015	A	0.02	0.19	F
5	rs10478424	118789020	*HSD17B4*	Randall 2013	A	0.04	0.78	F
5	rs6556301	176527577	*FGFR4*	Shungin 2015	T	0.02	0.36	-
6	rs7759742	32381736	*BTNL2*	Shungin 2015	A	0.02	0.51	-
6	rs1776897	34195011	*HMGA1*	Shungin 2015	G	0.03	0.08	F
6	rs6905288	43758873	*VEGFA*	Heid 2010	A	0.04	0.56	F
6	rs9491696	127452639	*RSPO3*	Heid 2010	G	0.04	0.48	F
7	rs1055144	25871109	*NFE2L3*	Heid 2010	T	0.04	0.21	-
7	rs1534696	26397239	*SNX10*	Shungin 2015	C	0.01	0.43	F
8	rs1550280	23601830	NKX2-6*	Shungin 2015	C	0.02	0.77	F
8	rs12679556	72514228	*MSC*	Shungin 2015	G	0.03	0.25	-
9	rs10991437	107735920	*ABCA1*	Shungin 2015	A	0.03	0.11	-
10	rs7917772	104487443	*SFXN2*	Shungin 2015	A	0.01	0.62	F
12	rs1443512	54342684	*HOXC13*^‡^	Heid 2010	A	0.03	0.24	F
12	rs4765219	124440110	*CCDC92*	Shungin 2015	C	0.03	0.67	-
15	rs8030605	56504598	*RFX7*	Shungin 2015	A	0.03	0.14	-
15	rs2165295	67027320	SMAD6*	Shungin 2015	A	0.02	0.71	-
16	rs2925979	81534790	*CMIP*	Shungin 2015	T	0.02	0.31	F
17	rs4646404	17420199	*PEMT*	Shungin 2015	G	0.03	0.67	-
18	rs12454712	60845884	*BCL2*	Shungin 2015	T	0.02	0.61	F
19	rs4531856	18388383	JUND*	Shungin 2015	C	0.02	0.36	-
19	rs4081724	33824946	*CEBPA*	Shungin 2015	G	0.04	0.85	-
20	rs979012	6623374	*BMP2*	Shungin 2015	T	0.03	0.34	-
20	rs224333	34023962	*GDF5*	Shungin 2015	G	0.02	0.62	M
20	rs6090583	45558831	*EYA2*	Shungin 2015	A	0.02	0.48	-
22	rs4823006	29451671	*ZNRF3*	Heid 2010	A	0.02	0.57	-

Where: WHRadjBMI, waist:hip ratio adjusted for body mass index calculated as per (Heid et al, 2010)

†, Locus name taken from Shungin et al; EA, reported effect or WHRadjBMI-increasing allele; β, reported effect size; reported EAF, effect allele frequency

*proxy used instead of reported tag SNP (rs1550280 instead of rs7830933, r^2^ = 0.96, rs2165295 instead of rs1440372, r^2^ = 1, rs4531856 instead of rs12608504, r^2^ = 1), but EAF given for the reported tag SNP

‡, same SNP reported by Heid et al but Beta and EAF given from Shungin et al

-, no evidence for sex-specific effects; F, evidence supports a stronger effect in women; M, evidence suggests a stronger effect in men.

** sex specific evidence

### Statistical Methods

Correlations between raw phenotypes were assessed by Spearman rank coefficients. All variables were assessed for normality and where a skewed distribution was observed, natural log transformed before further analysis. Statistical analyses were conducted in STATA (STATAcorp LP, Texas, USA). For genetic analysis, linear regression was used to assess the effect of SNPs on the traits of interest using additive models in PLINK [19]. As a sex effect was reported for most tagging SNPs, sex-specific analyses were conducted, as well as a sex-combined analysis including a SNP x sex interaction. Unweighted SNP scores were constructed by addition of the number of reported WHRadjBMI-increasing alleles (Table 2). Analyses of lipolysis measures were adjusted for sex, age, age^2^, and BMI as above for consistency and to enable comparison between phenotypes. Under the assumption that the heritability of lipolysis is similar to that of fat distribution [6], we have 99% power to detect an effect with nominal p value 0.05. False discovery rate (FDR) was calculated according to Benjamini-Hochberg. Because the selection of SNPs is based on known associations with related biological traits adjustment for multiple testing could be too conservative and a p value ≤0.05 is reported together with FDRs.

## Results

### Relationships between measures of obesity and lipolysis

In order to test our hypothesis that central obesity influences lipolysis we first examined whether measures of adipocyte lipolysis correlated with obesity measures. We investigated spontaneous, isoprenaline- (activating β-adrenoceptors) and dcAMP- (activating protein kinase A) stimulated lipolysis for correlations with the obesity phenotypes WHR, BMI and WHRadjBMI. All assessed obesity phenotypes correlated positively with spontaneous lipolysis but inversely with isoprenaline or dcAMP induced lipolysis (Table 3). As the tested variable most accurately reflecting the accumulation of metabolically detrimental fat independent of overall obesity [4], we focused on WHRadjBMI for further analyses.

**Table 3:** Spearman rank correlations of obesity phenotypes with adipocyte lipolysis measures.

	WHR			BMI			WHRadjBMI		
	N	Rho	P	N	Rho	P	N	Rho	P
Spontaneous lipolyis	461	0.267	<0.0001	470	0.398	<0.0001	461	0.157	0.0007
Isoprenaline-stimulated lipolysis*	710	-0.299	<0.0001	731	-0.319	<0.0001	710	-0.152	<0.0001
dcAMP-stimulated lipolysis*	712	-0.284	<0.0001	712	-0.296	<0.0001	712	-0.134	0.0003

Where: WHR, waist:hip ratio; WHRadjBMI, WHR adjusted for BMI, calculated as per (Heid *et al*, *2010*); Spontaneous lipolysis, mmol/glycerol per 2 hours and 10^7^ cells; dcAMP, dibutyryl cyclic AMP

* divided by basal lipolysis measures.

### Effect of WHRadjBMI-associated SNPs on lipolysis

Whether altered lipolysis causes or results from central fat accumulation remains unclear. Here, a Mendelian randomisation experiment was performed to test causality by using SNPs tagging 40 loci robustly associated with WHRadjBMI [4–6]. If central obesity regulates lipolysis (increased spontaneous lipolysis and/or reduced stimulated lipolysis) then it would be expected that SNPs robustly associated with increased central obesity would demonstrate consistent effects on lipolysis phenotypes. The WHRadjBMI-increasing allele previously reported [4–6] is taken as the true WHRadjBMI-increasing allele, and is referred to as such in the further analyses. The SNP scores were calculated by summing the number of WHRadjBMI-increasing alleles, and thus represent the genetic burden or predisposition to increased central obesity. Only subjects successfully genotyped for all SNPs included in the score were considered in the analysis. The SNP scores including all 40 tagging SNPs demonstrated no associations with all tested measures of lipolysis (Table 4). To exclude pleiotropic effects, a SNP score was calculated excluding SNPs (rs17451107, rs1195252, rs2925979, rs303084, rs4765219, rs4846567, rs9687846 and rs998584) with known effects on related traits, namely levels of HDL, LDL, TG or adiponectin and birthweight, which also revealed no associations with lipolysis measures (Table 4). It should be noted that for a larger cohort of 331–556 subjects (depending on phenotype) in which we assessed scores for the 14 loci known [4–5] prior to the recent Shungin *et al* report [6], we found a similar lack of association with the adipose lipolysis measures (S2 Table).

**Table 4:** Linear regression analysis of the effect of the WHRadjBMI-increasing SNPscore on lipolysis phenotypes.

	40SNPscore								40SNPscore no pleiotropy**							
	N	adjR2	Beta	Se	L95	U95	P_main_	P_inter_	N	adjR2	Beta	Se	L95	U95	P_main_	P_inter_
Spontaneous lipolysis	174	0.23	0.012	0.010	-0.008	0.032	0.2370	0.7800	174	0.22	0.006	0.011	-0.016	0.028	0.6090	0.8080
Isoprenaline-stimulated lipolysis*	305	0.17	-0.001	0.004	-0.009	0.006	0.7010	0.2290	305	0.17	0.001	0.004	-0.007	0.009	0.7860	0.1480
dcAMP-stimulated lipolysis*	294	0.15	-0.004	0.004	-0.013	0.004	0.2870	0.2140	294	0.14	-0.002	0.005	-0.011	0.007	0.6270	0.1680

Where: adjR2, adjusted R2 from regression models; L95, lower boundry of 95% confidence interval; U95, upper boundry of 95% confidence interval; Pmain, P value for main effect; Pinter, Pvalue for sex interaction

* compared to basal lipolysis levels

**SNP score excluding variants rs17451107, rs1195252, rs2925979, rs303084, rs4765219, rs4846567, rs9687846 and rs998584 which previously have been associated with related traits, namely levels of HDL, LDL, TG or adiponectin and birthweight

It is possible that some SNPs influence mechanisms leading to central fat accumulation but that their effects might be diluted when all SNPs are combined into the SNP score. Therefore we tested individual WHRadjBMI-associated SNPs for influence on measures of spontaneous and stimulated lipolysis (Table 5). Nominally significant associations with spontaneous lipolysis were detected for two loci in which the WHRadjBMI-increasing allele was associated with an increased rate of lipolysis (*PLXND1* rs10804591 and *CMIP* rs2925979). Moreover, two loci demonstrated effects on isoprenaline-stimulated lipolysis, with the WHRadjBMI-increasing allele being associated with increased lipolysis (*VEGFA* rs6905288 and *ZNRF3-KREMEN1* rs4823006). Finally, the WHRadjBMI-increasing allele of rs4823006 (*ZNRF3-KREMEN1*) was associated with increased dcAMP-stimulated lipolysis, whilst the WHRadjBMI-increasing allele of rs2925979 (*CMIP*) was associated with decreased dcAMP-stimulated lipolysis. None of these genetic associations were significant with FDR <5% after adjustment for the analysis of multiple SNPs (Table 5).

**Table 5:** Effect of individual SNPs tagging central obesity-associated loci on adipocyte lipolysis phenotypes.

	* *		Spontaneous lipolysis (n = 340–444)						Isoprenaline-stimulated lipolysis (n = 492–713)						dcAMP-stimulated lipolysis (n = 528–690)					
SNP	Locus	EA	BETA	SE	L95	U95	P	FDR	BETA	SE	L95	U95	P	FDR	BETA	SE	L95	U95	P	FDR
rs984222	*TBX15-WARS2*	G	0.05	0.04	-0.03	0.12	0.2172	0.95	-0.02	0.02	-0.05	0.01	0.1593	0.62	-0.03	0.02	-0.06	0.01	0.1187	0.68
rs1011731	*DNM3-PIGC*	G	-0.01	0.04	-0.09	0.06	0.7216	0.95	-0.02	0.02	-0.05	0.01	0.1497	0.62	-0.03	0.02	-0.06	0.00	0.0623	0.50
rs4846567	*LYPLAL1*	G	0.00	0.04	-0.08	0.08	0.9480	0.95	0.00	0.02	-0.04	0.03	0.7836	0.90	0.01	0.02	-0.02	0.04	0.5782	0.90
rs1385167	*MEIS1*	G	-0.01	0.06	-0.13	0.11	0.8921	0.95	0.02	0.02	-0.01	0.06	0.2026	0.62	0.02	0.02	-0.02	0.06	0.3849	0.90
rs10195252	*GRB14-COBLL1*	T	-0.02	0.04	-0.10	0.06	0.5957	0.95	0.01	0.02	-0.02	0.04	0.4279	0.89	0.00	0.02	-0.03	0.04	0.8408	0.94
rs4684854	*PPARG*	C	0.02	0.04	-0.05	0.10	0.5203	0.95	0.00	0.02	-0.03	0.03	0.8549	0.90	0.00	0.02	-0.03	0.03	0.9253	0.95
rs6784615	*PBRM1*	T	-0.06	0.09	-0.23	0.11	0.5103	0.95	0.05	0.03	-0.02	0.11	0.1866	0.62	0.04	0.04	-0.03	0.12	0.2802	0.86
rs6795735	*ADAMTS9*	C	0.01	0.04	-0.07	0.09	0.7640	0.95	-0.01	0.02	-0.03	0.02	0.7383	0.90	-0.01	0.02	-0.04	0.02	0.4749	0.90
rs10804591	*PLXND1*	A	**0.11**	**0.05**	**0.01**	**0.21**	**0.0381**	**0.76**	-0.02	0.02	-0.05	0.02	0.2898	0.72	-0.02	0.02	-0.05	0.02	0.4128	0.90
rs17451107	*LEKR1*	T	0.00	0.05	-0.09	0.09	0.9927	0.97	0.00	0.02	-0.03	0.03	0.8721	0.90	-0.01	0.02	-0.04	0.03	0.7397	0.90
rs3805389	*NMU*	A	0.02	0.04	-0.07	0.11	0.6534	0.95	0.03	0.02	-0.01	0.06	0.1091	0.62	0.03	0.02	0.00	0.07	0.0525	0.50
rs9991328	*FAM13A*	T	0.05	0.05	-0.05	0.14	0.3350	0.95	-0.01	0.02	-0.04	0.02	0.6355	0.90	0.00	0.02	-0.03	0.04	0.8150	0.94
rs303084	*SPATA5-FGF2*	A	-0.07	0.06	-0.18	0.05	0.2549	0.95	-0.01	0.02	-0.04	0.03	0.6996	0.90	0.00	0.02	-0.04	0.04	0.9535	0.95
rs459193	*MAP3K1*	A	0.02	0.05	-0.08	0.12	0.6987	0.95	0.02	0.02	-0.01	0.05	0.1821	0.62	0.03	0.02	0.00	0.07	0.0788	0.53
rs11743303	*MAP3K1*	G	-0.01	0.05	-0.11	0.09	0.8383	0.95	-0.01	0.02	-0.05	0.03	0.6311	0.90	-0.01	0.02	-0.05	0.03	0.6181	0.90
rs3936510	*MAP3K1*	T	-0.04	0.06	-0.17	0.09	0.5277	0.95	0.01	0.02	-0.03	0.05	0.7599	0.90	0.01	0.02	-0.03	0.06	0.5553	0.90
rs9687846	*MAP3K1*	A	-0.01	0.06	-0.13	0.12	0.8833	0.95	0.01	0.02	-0.03	0.05	0.7907	0.90	0.01	0.02	-0.04	0.05	0.7447	0.90
rs10478424	*HSD17B4*	A	0.04	0.05	-0.05	0.14	0.3990	0.95	-0.02	0.02	-0.05	0.02	0.3540	0.79	-0.01	0.02	-0.05	0.02	0.4711	0.90
rs6556301	*FGFR4*	T	0.04	0.05	-0.05	0.13	0.3884	0.95	0.00	0.02	-0.03	0.03	0.8925	0.90	-0.01	0.02	-0.04	0.02	0.5429	0.90
rs7759742	*BTNL2*	A	-0.02	0.05	-0.11	0.07	0.6768	0.95	-0.01	0.01	-0.04	0.02	0.5277	0.90	-0.02	0.02	-0.05	0.01	0.2154	0.84
rs1776897	*HMGA1*	G	-0.06	0.08	-0.22	0.11	0.4838	0.95	-0.01	0.03	-0.07	0.04	0.6295	0.90	0.00	0.03	-0.06	0.06	0.9153	0.95
rs6905288	*VEGFA*	A	-0.01	0.04	-0.09	0.07	0.8465	0.95	**0.03**	**0.01**	**0.01**	**0.06**	**0.0196**	**0.62**	0.02	0.02	-0.01	0.05	0.1541	0.69
rs9491696	*RSPO3*	G	0.05	0.04	-0.03	0.13	0.2341	0.95	-0.03	0.02	-0.06	0.00	0.0621	0.62	-0.03	0.02	-0.06	0.00	0.0583	0.50
rs1055144	*NFE2L3*	T	0.02	0.05	-0.08	0.12	0.7054	0.95	0.02	0.02	-0.02	0.05	0.4430	0.89	0.01	0.02	-0.03	0.05	0.5130	0.90
rs1534696	*SNX10*	C	-0.04	0.04	-0.12	0.04	0.3427	0.95	0.00	0.02	-0.03	0.03	0.8590	0.90	-0.01	0.02	-0.04	0.02	0.6175	0.90
rs1550280	*NKX2-6*	C	-0.05	0.05	-0.16	0.05	0.3154	0.95	0.02	0.02	-0.02	0.05	0.2848	0.72	0.02	0.02	-0.02	0.05	0.3694	0.90
rs12679556	*MSC*	G	-0.04	0.05	-0.15	0.07	0.4932	0.95	0.00	0.02	-0.03	0.04	0.7905	0.90	0.01	0.02	-0.03	0.04	0.7186	0.90
rs10991437	*ABCA1*	A	0.08	0.06	-0.04	0.21	0.1917	0.95	-0.01	0.02	-0.05	0.03	0.6192	0.90	-0.01	0.02	-0.05	0.04	0.7374	0.90
rs7917772	*SFXN2*	A	0.00	0.05	-0.09	0.10	0.9179	0.95	0.00	0.02	-0.03	0.03	0.9946	0.99	-0.01	0.02	-0.05	0.02	0.3890	0.90
rs1443512	*HOXC13*	A	0.01	0.04	-0.07	0.10	0.7438	0.95	0.02	0.02	-0.02	0.05	0.3116	0.73	0.01	0.02	-0.03	0.04	0.6510	0.90
rs4765219	*CCDC92*	C	-0.01	0.05	-0.10	0.08	0.8835	0.95	-0.02	0.02	-0.05	0.01	0.1798	0.62	-0.01	0.02	-0.04	0.02	0.4978	0.90
rs8030605	*RFX7*	A	-0.11	0.07	-0.26	0.04	0.1394	0.95	-0.03	0.02	-0.08	0.01	0.1629	0.62	-0.03	0.02	-0.08	0.02	0.2314	0.84
rs2165295	*SMAD6*	A	-0.03	0.05	-0.13	0.07	0.5424	0.95	0.02	0.02	-0.01	0.05	0.2303	0.66	0.02	0.02	-0.01	0.06	0.1552	0.69
rs2925979	*CMIP*	T	**0.12**	**0.05**	**0.03**	**0.22**	**0.0092**	**0.37**	-0.03	0.02	-0.06	0.00	0.0596	0.62	**-0.04**	**0.02**	**-0.07**	**-0.01**	**0.0125**	**0.33**
rs4646404	*PEMT*	G	-0.03	0.04	-0.10	0.04	0.4139	0.95	0.01	0.01	-0.02	0.03	0.6852	0.90	0.00	0.02	-0.03	0.03	0.9485	0.95
rs12454712	*BCL2*	T	0.04	0.04	-0.05	0.13	0.3897	0.95	0.00	0.01	-0.03	0.03	0.9244	0.90	0.01	0.02	-0.02	0.04	0.5459	0.90
rs4531856	*JUND*	C	0.06	0.05	-0.04	0.15	0.2243	0.95	0.01	0.02	-0.02	0.04	0.6787	0.90	0.00	0.02	-0.03	0.03	0.9407	0.95
rs4081724	*CEBPA*	G	0.00	0.07	-0.13	0.13	0.9800	0.97	0.01	0.02	-0.03	0.06	0.5820	0.90	0.00	0.02	-0.04	0.05	0.8471	0.94
rs979012	*BMP2*	T	-0.07	0.05	-0.16	0.02	0.1107	0.95	-0.03	0.02	-0.06	0.00	0.0969	0.62	-0.02	0.02	-0.05	0.01	0.2619	0.86
rs224333	*GDF5*	G	0.01	0.05	-0.08	0.10	0.8289	0.95	0.00	0.01	-0.03	0.03	0.9010	0.90	-0.01	0.02	-0.04	0.02	0.6267	0.90
rs6090583	*EYA2*	A	-0.01	0.05	-0.10	0.08	0.8211	0.95	0.00	0.02	-0.03	0.04	0.7791	0.90	0.00	0.02	-0.03	0.03	0.9455	0.95
rs4823006	*ZNRF3-KREMEN1*	A	0.00	0.04	-0.08	0.08	0.9934	0.99	**0.03**	**0.02**	**0.00**	**0.06**	**0.0360**	**0.62**	**0.04**	**0.02**	**0.01**	**0.07**	**0.0166**	**0.33**

Where: EA, effect (WHRadjBMI-increasing) allele; L95, lower boundry of 95% confidence interval; U95, upper boundry of 95% confidence interval; analyses adjusted for sex, age, age^2^ and BMI; FDR, false discovery rate

### Influence of individual WHR-adjBMI-associated genetic variants on central obesity

To determine whether the SNP effects on WHRadjBMI in our study concurred with those reported previously, linear regression analyses was conducted. Of 40 SNPs analysed here, 24 of the reported WHRadjBMI-increasing alleles demonstrated the expected positive effect on WHRadjBMI in this cohort, and 7 SNPs demonstrated the expected effect size (Table 6) [4–6]. For most complex quantitative traits studied to date, maximum effect sizes of ~0.05 have been reported. Thus, the effect sizes of 32 SNPs, even if differing slightly from those previously reported, are unsurprising whilst the 8 SNPs with effect sizes larger than 0.05 should be interpreted with caution (Table 6) [4–6]. These inconsistencies might be due to the relative small sample size of subjects available here (n = approximately 750 compared to the reported n = 210,000 [6]), the enrichment for obese subjects in the here analysed study population, or the relative numbers of men and women (n = 202 and n = 587 respectively). The male-specific analysis shows effect sizes rather larger than those previously observed [6], but the relatively small sample size (n = 202) means that this is likely a spurious result.

**Table 6:** Effect of SNPs tagging central obesity-associated loci on WHRadjBMI.

	* *		All subjects (n = 516–739)					Women (n = 425–558)					Men (n = 142–181)					P_inter_
SNP	Locus	EA	Beta	Se	L95	U95	P	Beta	Se	L95	U95	P	Beta	Se	L95	U95	P	
rs984222†	*TBX15-WARS2*	G	0.07	0.06	-0.04	0.18	0.2238	0.08	0.07	-0.05	0.21	0.2551	0.06	0.11	-0.16	0.28	0.5831	0.8411
rs1011731†*	*DNM3-PIGC*	G	0.03	0.06	-0.08	0.14	0.6106	0.01	0.07	-0.11	0.14	0.8213	0.06	0.11	-0.15	0.27	0.5680	0.7299
rs4846567†	*LYPLAL1*	G	**0.13**	**0.06**	**0.02**	**0.25**	**0.0209**	**0.21**	**0.07**	**0.07**	**0.34**	**0.0025**	-0.08	0.11	-0.29	0.14	0.4708	**0.0273**
rs1385167	*MEIS1*	G	-0.01	0.07	-0.15	0.13	0.8669	-0.06	0.08	-0.21	0.10	0.4774	0.16	0.15	-0.14	0.45	0.3051	0.2118
rs10195252	*GRB14-COBLL1*	T	0.00	0.06	-0.11	0.12	0.9478	0.00	0.07	-0.13	0.13	0.9898	0.02	0.11	-0.19	0.23	0.8493	0.9264
rs4684854†	*PPARG*	C	0.02	0.06	-0.09	0.13	0.6948	0.09	0.06	-0.04	0.21	0.1610	**-0.25**	**0.12**	**-0.48**	**-0.02**	**0.0343**	**0.0138**
rs6784615†*	*PBRM1*	T	0.04	0.13	-0.21	0.29	0.7480	-0.07	0.14	-0.35	0.21	0.6325	0.46	0.29	-0.10	1.02	0.1118	0.0834
rs6795735	*ADAMTS9*	C	-0.01	0.06	-0.12	0.10	0.7964	0.01	0.06	-0.12	0.14	0.8839	-0.08	0.11	-0.30	0.14	0.4650	0.5088
rs10804591†	*PLXND1*	A	0.12	0.07	-0.01	0.25	0.0687	**0.17**	**0.08**	**0.02**	**0.32**	**0.0258**	0.01	0.12	-0.23	0.26	0.9153	0.2089
rs17451107†	*LEKR1*	T	0.02	0.06	-0.09	0.13	0.7508	0.02	0.06	-0.11	0.14	0.7758	0.04	0.11	-0.17	0.25	0.7044	0.8555
rs3805389†	*NMU*	A	0.02	0.06	-0.10	0.14	0.7531	-0.05	0.07	-0.20	0.09	0.4632	0.24	0.13	-0.01	0.48	0.0660	**0.0471**
rs9991328†	*FAM13A*	T	0.04	0.05	-0.06	0.15	0.4324	0.05	0.06	-0.08	0.17	0.4727	0.03	0.11	-0.18	0.24	0.7749	0.7202
rs303084†	*SPATA5-FGF2*	A	0.01	0.07	-0.12	0.14	0.8995	0.04	0.08	-0.11	0.19	0.5795	-0.09	0.14	-0.37	0.18	0.5006	0.3785
rs11743303†	*MAP3K1*	G	0.07	0.07	-0.08	0.21	0.3613	0.01	0.09	-0.16	0.19	0.8839	0.15	0.13	-0.11	0.41	0.2539	0.2706
rs9687846*	*MAP3K1*	A	-0.02	0.07	-0.17	0.12	0.7576	-0.06	0.09	-0.23	0.11	0.5197	0.02	0.14	-0.25	0.29	0.8889	0.5133
rs10478424	*HSD17B4*	A	-0.03	0.07	-0.17	0.10	0.6213	0.06	0.08	-0.09	0.22	0.4145	**-0.37**	**0.14**	**-0.64**	**-0.10**	**0.0083**	**0.0082**
rs6556301†	*FGFR4*	T	0.01	0.05	-0.09	0.12	0.8117	-0.03	0.06	-0.15	0.10	0.6838	0.17	0.11	-0.04	0.38	0.1233	0.1179
rs7759742†*	*BTNL2*	A	0.02	0.05	-0.09	0.12	0.7580	0.07	0.06	-0.04	0.19	0.2246	-0.16	0.10	-0.35	0.04	0.1148	0.0809
rs1776897†	*HMGA1*	G	0.17	0.10	-0.03	0.38	0.0921	0.09	0.12	-0.15	0.33	0.4545	0.37	0.19	0.00	0.73	0.0537	0.1932
rs6905288†	*VEGFA*	A	0.03	0.06	-0.08	0.14	0.5458	0.05	0.07	-0.08	0.18	0.4494	0.00	0.11	-0.21	0.21	0.9842	0.6982
rs9491696†	*RSPO3*	G	0.01	0.06	-0.10	0.12	0.8768	0.02	0.07	-0.11	0.15	0.7881	0.00	0.10	-0.21	0.20	0.9953	0.8313
rs1534696*	*SNX10*	C	-0.01	0.06	-0.13	0.10	0.8280	-0.01	0.07	-0.14	0.12	0.9015	-0.03	0.12	-0.27	0.22	0.8296	0.8925
rs1055144†*	*NFE2L3*	T	0.04	0.07	-0.10	0.18	0.5450	0.06	0.08	-0.10	0.22	0.4716	-0.04	0.15	-0.34	0.26	0.8074	0.6040
rs1550280*	*NKX2-6*	C	-0.02	0.06	-0.14	0.11	0.7898	0.01	0.07	-0.13	0.15	0.9223	-0.05	0.13	-0.31	0.20	0.6758	0.4796
rs12679556†	*MSC*	G	0.11	0.06	-0.01	0.24	0.0775	0.11	0.07	-0.04	0.26	0.1377	0.14	0.12	-0.10	0.39	0.2477	0.7434
rs10991437	*ABCA1*	A	-0.01	0.08	-0.16	0.14	0.9265	-0.06	0.09	-0.24	0.11	0.4856	0.19	0.14	-0.10	0.47	0.1960	0.1407
rs7917772†	*SFXN2*	A	0.04	0.06	-0.07	0.15	0.4429	0.03	0.07	-0.10	0.16	0.6470	0.02	0.11	-0.19	0.23	0.8552	0.7680
rs1443512	*HOXC13*	A	-0.01	0.06	-0.13	0.12	0.9304	0.01	0.07	-0.13	0.16	0.8776	-0.05	0.13	-0.31	0.20	0.6791	0.7217
rs4765219†*	*CCDC92*	C	0.03	0.06	-0.08	0.14	0.6315	0.04	0.06	-0.08	0.17	0.4897	-0.01	0.11	-0.22	0.20	0.9266	0.6091
rs8030605	*RFX7*	A	-0.06	0.08	-0.22	0.10	0.4367	-0.02	0.09	-0.20	0.16	0.7990	-0.17	0.18	-0.52	0.19	0.3557	0.3421
rs2165295	*SMAD6*	A	-0.03	0.06	-0.14	0.08	0.6061	0.00	0.07	-0.14	0.13	0.9780	-0.13	0.11	-0.34	0.08	0.2326	0.4241
rs2925979†	*CMIP*	T	0.04	0.05	-0.07	0.15	0.4459	0.09	0.06	-0.04	0.22	0.1651	-0.07	0.10	-0.27	0.13	0.4863	0.1259
rs4646404	*PEMT*	G	**-0.12**	**0.06**	**-0.23**	**-0.01**	**0.0305**	**-0.13**	**0.07**	**-0.26**	**0.00**	**0.0461**	-0.09	0.10	-0.29	0.11	0.3696	0.7364
rs12454712	*BCL2*	T	-0.03	0.05	-0.13	0.07	0.5588	-0.03	0.06	-0.15	0.09	0.6689	-0.10	0.10	-0.30	0.10	0.3181	0.7877
rs4531856	*JUND*	C	-0.01	0.05	-0.12	0.10	0.8775	0.02	0.06	-0.10	0.14	0.7216	-0.16	0.12	-0.38	0.07	0.1799	0.3273
rs4081724†	*CEBPA*	G	0.05	0.08	-0.11	0.21	0.5480	0.06	0.09	-0.13	0.24	0.5457	-0.02	0.15	-0.32	0.28	0.9015	0.9190
rs979012†	*BMP2*	T	0.01	0.06	-0.10	0.12	0.8866	-0.01	0.07	-0.14	0.12	0.8766	0.09	0.11	-0.12	0.29	0.4144	0.5297
rs224333	*GDF5*	G	-0.03	0.05	-0.13	0.08	0.6297	-0.06	0.06	-0.18	0.06	0.3179	0.09	0.11	-0.12	0.30	0.4072	0.1354
rs6090583	*EYA2*	A	-0.02	0.06	-0.13	0.09	0.7381	0.01	0.06	-0.11	0.14	0.8178	-0.09	0.12	-0.31	0.14	0.4667	0.4072
rs4823006	*ZNRF3-KREMEN1*	A	-0.03	0.06	-0.14	0.08	0.5498	-0.02	0.07	-0.15	0.11	0.7787	-0.07	0.11	-0.28	0.14	0.5242	0.6126

Where: EA, effect (WHRadjBMI-increasing) allele thus a positive beta is consistent with the previous publications; L95, lower boundry of 95% confidence interval; U95, upper boundry of 95% confidence interval

*, effects sizes the same as those previously reported

†, effect directions consistent with those previously reported (Shungin et al, 2015). Analyses adjusted for sex, age, age^2^ and BMI. Pinter, P for sex interaction.

## Discussion

Whilst a plethora of genetic variants associated with measures of obesity have been identified, the modulated molecular mechanisms underpinning poor metabolic regulation remain elusive. WHR, when adjusted for BMI (WHRadjBMI [4]), has been proposed as representative of central fat accumulation independent of total obesity. Here we used WHRadjBMI-associated loci to investigate whether genetically determined central fat accumulation controls lipolysis functions of abdominal subcutaneous adipocytes. If central fat accumulation is driving the spontaneous or stimulated adipocyte lipolysis, then the SNPs robustly associated with central fat accumulation would be expected to have a similar effect on lipolysis. Whilst the SNP score analysis does not fulfil the criteria for a true Mendelian randomisation experiment (because the WHRadjBMI-associated SNPs do not show the expected associations with WHRadjBMI), it does provide novel evidence to suggest that, generally speaking, genetically-determined central fat accumulation has little effect on adipocyte lipolysis. A direct test of the reverse hypothesis that adipose lipolysis capacity determines central fat accumulation is unfortunately not yet possible.

The nominal associations between specific tagging SNPs identified by WHRadjBMI GWAS and lipolysis measures reported here, although becoming nonsignificant after adjustment for multiple testing. highlights potential novel biology as none of the candidate genes suggested by Shungin et al (6) for these loci are established regulators of lipolysis [20].

Firstly, effects on NFκB signalling. CMIP has been reported to influence NFκB signalling [21] which in turn can modulate adipocyte lipolysis [22]. Recently our group has demonstrated that *CMIP* expression levels were negatively associated with insulin-stimulated lipogenesis in subcutaneous adipocytes [23]. The observation here that the rs2925979 *CMIP* locus is also associated with spontaneous and dcAMP-stimulated lipolysis extends mechanistic understanding of this locus.

Secondly, effects on adipocyte morphology. *PLXND1* has been shown to regulate adipose morphology, i.e. the relationship between adipocyte size and number (24), with a genetic deficiency demonstrating reduced fat mass [24]. In light of the fact that spontaneous lipolysis is strongly correlated with adipocyte size, as previously discussed [10], the WHRadjBMI-increasing allele of rs10804591 in the *PLXND1* locus demonstrating a positive association with increased spontaneous lipolysis is intriguing. Furthermore, the WHRadjBMI-increasing allele of rs6905288 in the *VEGFA* locus revealed a positive correlation with isoprenaline-stimulated lipolysis. Interestingly, expression of VEGFA in adipose tissue has been shown to have beneficial metabolic effects via proangiogenic activity during adipose tissue expansion [25].

As for *KREMEN1* and *ZNRF3*, associated here with both dcAMP- and isoprenaline-stimulated lipolysis, both are involved in Wnt signalling, which inhibits adipogenesis [26–28]. Considering that that WHRadjBMI is inversely associated with stimulated lipolysis, it is surprising that the WHRadjBMI increasing allele at the *VEGFA* and *KREMEN1*-*ZNRF3* loci are associated with increased stimulated lipolysis. This raises the question whether these are spurious associations.

Whilst the cohort studied here is unique regarding the relatively large number of subjects with specific adipocyte lipolysis measures, there are some limitations to this study. Incomplete genotyping, finally including 40 of 49 reported WHRadjBMI-associated loci, means that the SNP score does not fully represent genetically-determined fat distribution. Also, for some SNPs we cannot confirm reported GWAS associations with WHRadjBMI [6], adding some uncertainty to our conclusion that WHRadjBMI does not regulate adipose lipolysis. The nominal genetic associations reported here were became nonsignificant after adjustment for multiple testing and would therefore benefit form replication in an independent cohort. Unfortunately there is as far as we know no replication cohort available. Despite the shortfalls of this study, we believe that the findings are relevant since we used established genetic associations to select the SNPs and based the study on a hypothesis that was experimentally validated, i.e. the association reported here between measures of obesity and lipolysis.

In summary, this study shows nominal significant associations between specific central obesity loci and measures of lipolysis functions in abdominal subcutaneous adipose tissue but refutes genetically increased central fat accumulation as a main cause of altered lipolysis. Whilst generally weak associations were observed, our findings suggest that the mechanisms by which genetic variants influence central fat accumulation are diverse and locus-specific.

## Supporting Information

S1 Table Genotypes of SNPs failing the Hardy-Weinberg equilibrium test. (DOCX)

S2 Table Linear regression analysis of the effect of the smaller WHRadjBMI-increasing SNPscore on lipolysis phenotypes. (DOCX)

S3 Table Genotype data. (XLSX)

S1 Fig Schematic demonstrating the different steps by which isoprenaline and dibutyryl cyclic AMP (dcAMP) stimulate activation of lipolysis to produce release of glycerol. In blue, endogenous cellular components, in red the 2 stimuli used in this study. Isoprenaline-stimulated activation relies upon the efficient function of the β-adrenergic receptor signalling to activation of protein kinase A. In contrast, dcAMP directly activates Protein kinase A to initiate glycerol release.
(DOCX)
