# Supplementary material for: Effects of Genetic Loci Associated with Central Obesity on Adipocyte Lipolysis
Source: PLoS One. 2016 Apr 22;11(4):e0153990. doi: 10.1371/journal.pone.0153990 (PMC4841524; doi:10.1371/journal.pone.0153990)
Supplement: S1 Table — (DOCX) [file pone.0153990.s001.docx]

**Supporting information**

**S1 Table**: Genotypes of SNPs failing the Hardy-Weinberg equilibrium test

| CHR | SNP | EA | Homozygous variant | Heterozygous | Homozygous common | O(HET) | E(HET) | P | observed EAF | reported EAF |
| --- | --- | --- | --- | --- | --- | --- | --- | --- | --- | --- |
| 4 | rs3805389 | A | 54 | 200 | 341 | 0.3361 | 0.3837 | 0.002782 | 0.2588 | 0.012 |
| 6 | rs1294421 | C | 260 | 24 | 379 | 0.0362 | 0.4839 | **6.36E-152** | 0.4103 | 0.031 |
| 7 | rs1534696 | C | 109 | 207 | 226 | 0.3819 | 0.4767 | 4.04E-06 | 0.3921 | 0.011 |
| 12 | rs718314 | G | 160 | 127 | 376 | 0.1916 | 0.4469 | **4.37E-49** | 0.3371 | 0.259 |
| 17 | rs4646404 | A | 92 | 107 | 448 | 0.1654 | 0.3486 | 4.22E-36 | 0.2249 | 0.027 |
| Where: EA, effect or WHRadjBMI-increasing allele; β, effect size; EAF, effect allele frequency; Bold indicates possible technical error. | | | | | | | | | | |
