# Supplementary material for: Effects of Genetic Loci Associated with Central Obesity on Adipocyte Lipolysis
Source: PLoS One. 2016 Apr 22;11(4):e0153990. doi: 10.1371/journal.pone.0153990 (PMC4841524; doi:10.1371/journal.pone.0153990)
Supplement: S2 Table — (DOCX) [file pone.0153990.s002.docx]

**S2 Table**: Linear regression analysis of the effect of the smaller (pre Shungin et al, 2015 publication) WHRadjBMI-increasing SNPscore on lipolysis phenotypes.

|  | pre Shungin SNP score | | | | | | | | pre Shungin SNP score no pleiotropy | | | | | | | |
| --- | --- | --- | --- | --- | --- | --- | --- | --- | --- | --- | --- | --- | --- | --- | --- | --- |
|  | N | adjR2 | Beta | Se | L95 | U95 | P_main_ | P_inter_ | N | adjR2 | Beta | Se | L95 | U95 | P_main_ | P_inter_ |
| Spontaneous lipolysis | 322 | 0.321 | 0.008 | 0.012 | -0.015 | 0.031 | 0.4850 | 0.3130 | 322 | 0.323 | 0.014 | 0.013 | -0.012 | 0.040 | 0.2960 | 0.5780 |
| Isoprenaline-stimulated lipolysis* | 551 | 0.182 | 0.000 | 0.004 | -0.009 | 0.009 | 0.9920 | 0.3400 | 551 | 0.182 | -0.001 | 0.005 | -0.011 | 0.009 | 0.8230 | 0.5610 |
| dcAMP-stimulated lipolysis* | 534 | 0.163 | -0.002 | 0.005 | -0.012 | 0.007 | 0.6450 | 0.8880 | 534 | 0.163 | -0.004 | 0.005 | -0.015 | 0.006 | 0.4380 | 0.6650 |
| Where: adjR2, adjusted R2 from regression models; L95, lower boundry of 95% confidence interval; U95, upper boundry of 95% confidence interval; Pmain, P value for main effect; Pinter, Pvalue for sex interaction; * compared to basal lipolysis levels. The limited score based upon 14 lead SNPs reported by Randall et al and Heid et al (*TBX15-WARS2, DNM3, LYPLAL1, GRB14-COBBL1, PPARG, NISCH-STAB, ADAMTS9, MAP3K1, HSD17B4, VEGFA, RSPO3, NFE2L3, HOXC13, ZNRF3-KREMEN1* loci). | | | | | | | | | | | | | | | | |

**S1 Figure 1: Schematic demonstrating the different steps by which isoprenaline and dibutyryl cyclic AMP (dcAMP) stimulate activation of lipolysis to produce release of glycerol.** In blue, endogenous cellular components, in red the 2 stimuli used in this study. Isoprenaline-stimulated activation relies upon the efficient function of the β-adrenergic receptor signalling to activation of protein kinase A. In contrast, dcAMP directly activates Protein kinase A to initiate glycerol release.

**
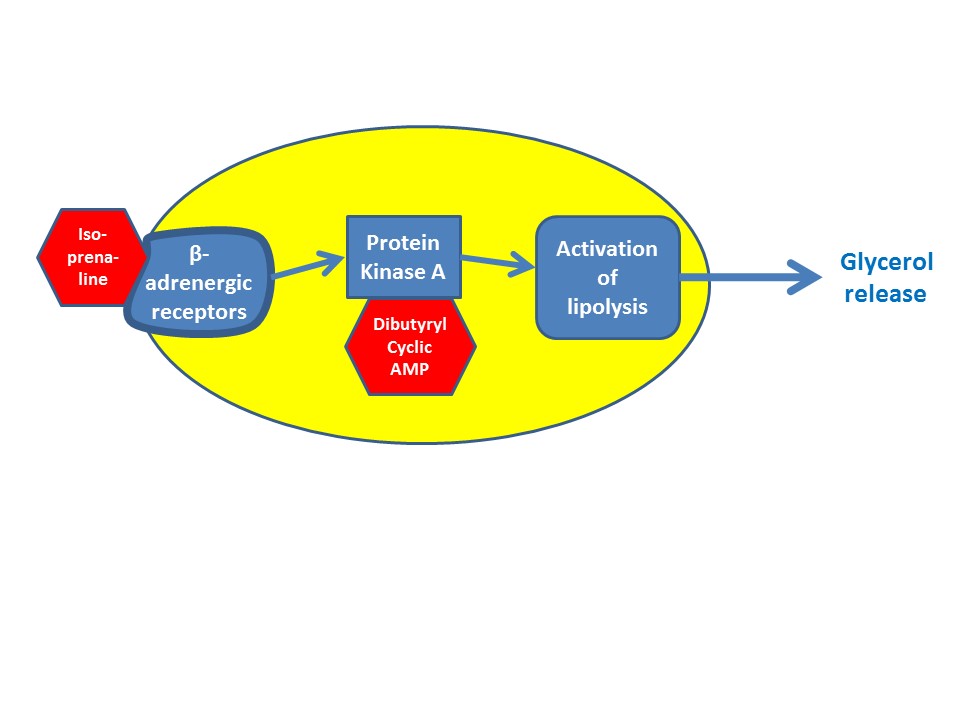
**
