# Supplementary material for: Effects of Genetic Loci Associated with Central Obesity on Adipocyte Lipolysis
Source: PLoS One. 2016 Apr 22;11(4):e0153990. doi: 10.1371/journal.pone.0153990 (PMC4841524; doi:10.1371/journal.pone.0153990)
Supplement: S1 Fig — In blue, endogenous cellular components, in red the 2 stimuli used in this study. Isoprenaline-stimulated activation relies upon the efficient function of the β-adrenergic receptor signalling to activation of protein kinase A. In contrast, dcAMP directly activates Protein kinase A to initiate glycerol release. (DOCX) [file pone.0153990.s004.docx]

**S1 Figure: Schematic demonstrating the different steps by which isoprenaline and dibutyryl cyclic AMP (dcAMP) stimulate activation of lipolysis to produce release of glycerol.** In blue, endogenous cellular components, in red the 2 stimuli used in this study. Isoprenaline-stimulated activation relies upon the efficient function of the β-adrenergic receptor signalling to activation of protein kinase A. In contrast, dcAMP directly activates Protein kinase A to initiate glycerol release.

**
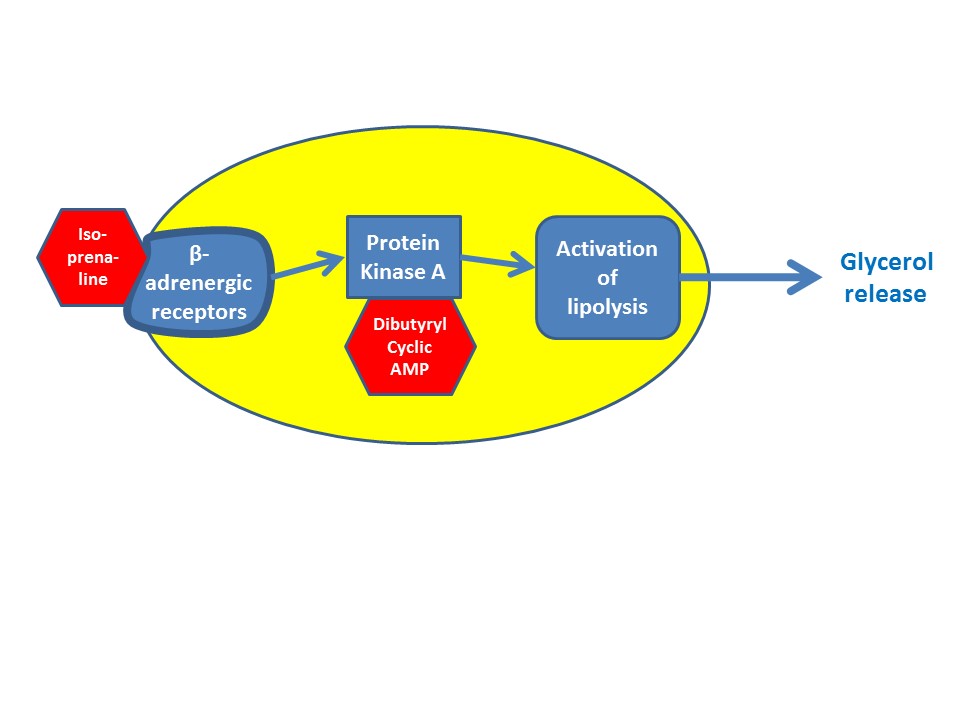
**
